# Short-Term Statistical Learning Mitigates the Ill-Posed Problem of Sound Localization

**DOI:** 10.1177/23312165261465030

**Published:** 2026-07-09

**Authors:** Robert Baumgartner, Roberto Barumerli, Benedikt Brands, Piotr Majdak

**Affiliations:** 1 Acoustics Research Institute, 31390 Austrian Academy of Sciences, Vienna, Austria; 2 Dyson School of Design Engineering, Imperial College London, London, United Kingdom

**Keywords:** spectral-shape cues, head-related transfer function (HRTF), source priors, perceptual inference, perceptual learning

## Abstract

The dynamic interplay between source-specific spectral features and spatial cues is central to auditory inference. While sagittal-plane localization relies on direction-dependent spectral cues shaped by the listener’s anatomy, sound sources themselves introduce spectral patterns that can obscure these cues, creating an ill-posed inference problem. We tested whether listeners can mitigate that problem by statistically learning a source’s spectral shape over the short term. In a free-field localization task, participants localized ripple-spectrum sounds under two conditions: within a block, source spectra were either fixed (*predictable*) or randomized (*unpredictable*). Predictability reduced large-scale localization errors – such as front-back reversals and quadrant confusions – by up to 5% within minutes. These findings demonstrate that listeners exploit spectral consistency across stimulus history to adapt spatial decoding, providing empirical evidence for short-term updating of spectral priors and underscoring the adaptive nature of auditory inference.

## 1 Introduction

Auditory localization plays a crucial role in everyday life, from safely navigating our surroundings to experiencing immersive entertainment. It relies on interpreting multiple acoustic cues, including interaural differences in time of arrival and intensity, as well as monaural spectral-shape cues induced by the listener’s anatomy (for reviews, see Middlebrooks, 2015; van Opstal, 2016; Majdak et al., 2020). Interaural cues provide accurate estimates of source laterality but are largely ambiguous along the polar-angle dimension, making it difficult to distinguish front/back and up/down positions. Here, monaural spectral cues are particularly important for encoding sound source direction. The spectral shape arriving at the ear results from a multiplication of the source spectrum and the listener’s head-related transfer function (HRTF). Inferring source direction from this mixture is an ill-posed inverse problem because infinitely many combinations of source spectrum and HRTF can produce the same ear signal. Consequently, localization accuracy can depend on the source spectrum. For instance, severe degradations are known to occur for sounds with narrow bandwidth (Middlebrooks, 1992), irregular spectral scrambling (Martin et al., 2006; Wightman & Kistler, 1997) or more orderly spectral ripples (Macpherson & Middlebrooks, 2003). In contrast, localization performance seems to be rather robust in real life. Listeners must rely on additional assumptions about likely source spectra and environmental structure to achieve stable spatial percepts. How flexibly those assumptions are formed remains largely unknown.

Long-term experience provides part of the solution to the ill-posed problem. Evolutionary pressures may have shaped the outer ear anatomy to emphasize the frequency statistics of natural environments (Parise et al., 2014). Listeners learn the distinct spectral consequences of their own anatomy and can also incorporate new ones (Friedrich & Schönwiesner, 2025; Hofman et al., 1998). This constrains the inference problem to a restricted set of spatial cues. To further constrain the inference process from the side of the identity cues, listeners may also have established long-term priors of naturally occurring source characteristics. Consistent with this idea, early work from Plenge and Brunschen (1971) showed that listeners localized speech sounds from familiar talkers more accurately than those from unknown talkers. Recent Bayesian modeling work (Reijniers et al., 2025) further investigated this topic by simulating previous localization experiments with flat noise (Best et al., 2011) and ripple-spectrum noise (Macpherson & Middlebrooks, 2003). Their Bayesian observer was tested using different source priors, either a flat-spectrum prior perfectly targeted to the flat noise localization task and a multi-purpose prior estimated from large databases with samples of environmental sounds or speech. Better-than-human-performance of the model using the task-specific flat-spectrum prior led the authors to conclude that listeners may use a generic, multi-purpose spectral prior based on everyday stimulus exposure rather than adapting to the presented stimulus ensemble.

This hypothesized lack of short-term adaptation is surprising, given that listeners frequently encounter novel or rapidly changing sound spectra. Moreover, evidence shows that listeners can track the statistics of rapid sound sequences with near-optimal Bayesian performance. Such capabilities have been demonstrated across multiple perceptual domains, including pitch (Barascud et al., 2016; Sedley et al., 2016), horizontal space (Bayram et al., 2025; Krishnamurthy et al., 2017), and even combinations of the two (Skerritt-Davis & Elhilali, 2021). For vertical space, listeners also integrate preceding sensorimotor information to reduce localization errors, albeit in a somewhat suboptimal manner (Ege et al., 2019). Furthermore, Macpherson (1998) compared polar-angle localization across unpredictable and frozen scrambled spectra and found a modest benefit of predictability for the scrambled spectra, but not for all listeners. These findings suggest that processes should exist to establish momentarily refined spectral priors for improving sound localization based on spectral cues. Yet, direct empirical evidence is limited.

To address this gap, the present study investigates short-term statistical learning of the sound-source spectrum as a potential mechanism for resolving the ill-posed nature of sound localization. Specifically, we test whether – and how rapidly – the auditory system exploits recent spectral regularities to constrain spatial inference and mitigate ambiguity. To this end, we measured directional localization performance under two conditions: one in which the stimulus spectrum remained fixed across trials of a block (*predictable*) and another in which it varied randomly from trial to trial (*unpredictable*).

## 2 Methods

### 2.1 Participants

Eleven normal-hearing participants (six male, five female; age range: 24 to 33 years, mean: 28 years) were recruited. Hearing thresholds were within normal limits ( ≤20 dB HL) between 125 Hz and 12.5 kHz as assessed by pure-tone audiometry. Participants were experienced in localization experiments but unfamiliar with our specific paradigm. They provided informed consent and received monetary compensation for their time. The study was conducted in accordance with the ethics statement for human experimental research conducted at the Acoustics Research Institute of the Austrian Academy of Sciences, as approved by the Commission for Science Ethics of the Austrian Academy of Sciences on April 23, 2024.

### 2.2 Stimuli

Stimuli consisted of Gaussian white noise bursts of 250-ms duration. A flat-spectrum noise burst served as the reference for the comparison with literature and to check the general localization ability of a participant. The ripple-spectrum noise bursts were generated by manipulating the flat-spectrum bursts in the Fourier domain. The phase spectrum remained unchanged (re-applied at inverse Fourier transform) while the magnitude spectrum was replaced by a sinusoidal function (Macpherson & Middlebrooks, 2003):

$$S_{\text{dB}}\left(f\right)=\frac{A}{2}\sin\left(2\pi \cdot \rho \cdot \log_{2}\left(f\right)+\phi \right),$$

where *S*_dB_(*f*) denotes the desired magnitude spectrum (in dB), *f* denotes frequency, *A* denotes the ripple depth (peak-to-trough amplitude, in dB), *ρ* denotes the ripple density (in ripples/octave), and *ϕ* denotes the ripple phase. Ripples were created only within the frequency range from 1 kHz to 16 kHz while eighth octave wide squared cosine ramps were applied at the edges to avoid discontinuities. The magnitude spectrum was set to 0 dB from 0.6 kHz to 1 kHz and to −100 dB both below 0.6 kHz and above 16 kHz. Ripple depth and density were fixed at *A* = 40 dB and *ρ* = 1, respectively, to achieve maximum interference (Macpherson & Middlebrooks, 2003). Ripple phase was fixed at 0°, 45°, 90°, 135°, and 180° in order to test the effect of various phases and to average the effects of a specific ripple phase potentially interfering with the individual HRTFs. Figure 1 shows the magnitude spectra for applied ripple phases. These phases do not cover the full 360° range uniformly, so there are frequencies where a peak never appears and others where a notch never appears. The code to generate such ripple-spectrum noise bursts is implemented as sig_macpherson2003 in the Auditory Modeling Toolbox (AMT, Majdak et al., 2022).

**Figure 1.**
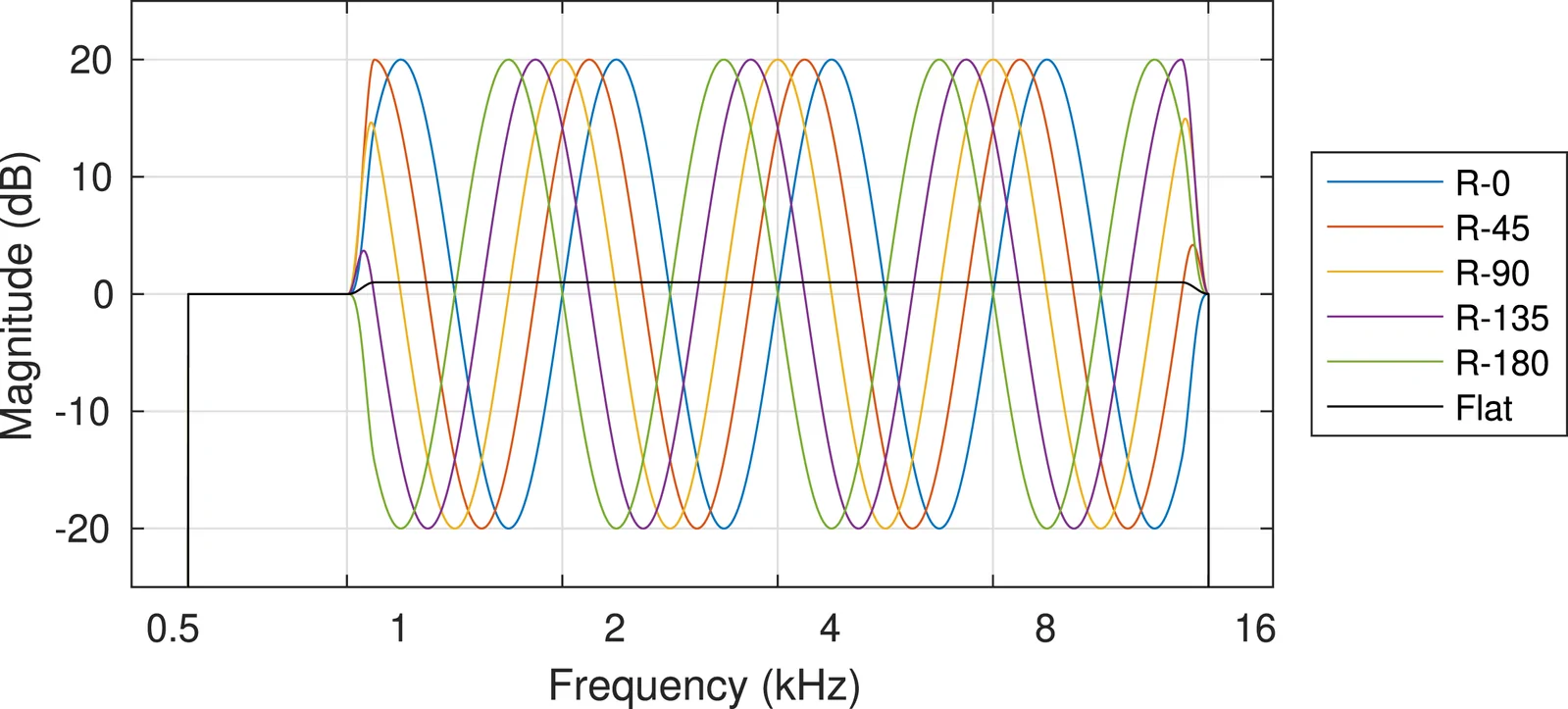
Spectral envelopes of the flat- and ripple-spectrum stimuli. The ripple types are labeled as ’R(ipple)-*ϕ*’ (with *ϕ* in degrees)

Afterwards stimuli were gated with 20-ms raised-cosine ramps at on- and offset. Ten realizations of each stimulus were generated and used in a random manner in order to prevent participants from learning specific spectral structures. Loudness was adjusted across stimulus types based on predictions of a short-term binaural loudness model (Moore et al., 2016) implemented in the AMT (Majdak et al., 2022). The sensation level of the stimuli was set to around 50 dB as determined for the frontal loudspeaker position using a manual one-up-two-down staircase procedure. Presentation levels were roved by ±3 dB to prevent localization strategies based on mere sound intensity.

### 2.3 Apparatus

Acoustic stimuli were presented via loudspeakers (E301, KEF) in a semi-anechoic room. The 91 loudspeakers were distributed over a spherical array with a radius of 1.2 m and elevation angles ranging from -47° to 90° (Figure 2). They were driven by four 24-channel amplifiers (d:24, Sonible). Digital-to-analog conversion was handled by an audio interface (MADIface XT, RME).

**Figure 2.**
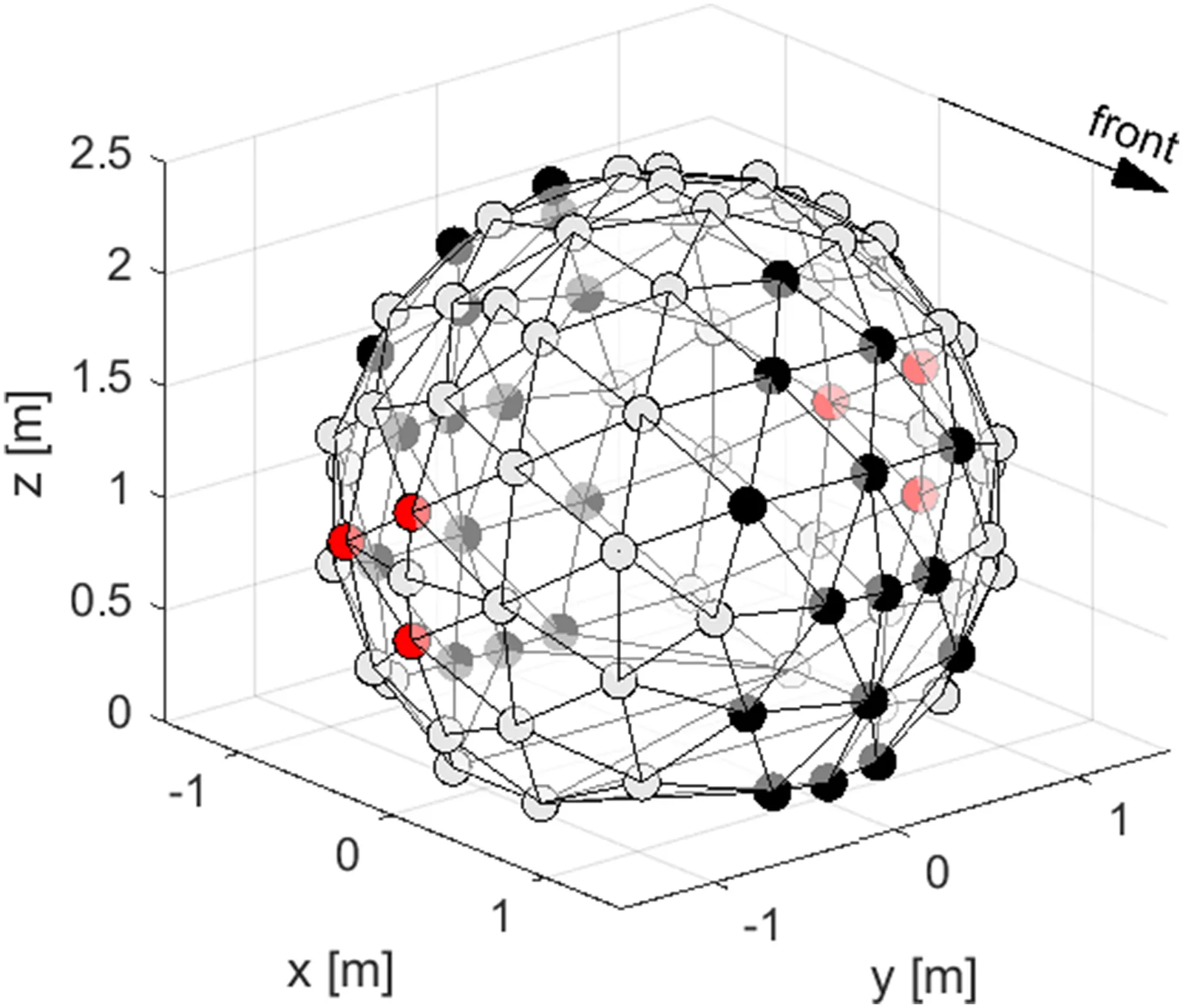
Loudspeaker positions in the spherical loudspeaker array. Black: Tested target positions around the median plane. Red: Lateral spread control positions around the interaural axis. White: Untested target positions only used during training

During sound localization experiments, the participants were sitting on a swivel chair mounted exactly in the center of the loudspeaker array. They were wearing a head-mounted display (HMD) and held a pointing device with a button in their hand. Both devices were tracked by three infrared cameras (Oculus Rift CV1, Meta), providing six degrees of freedom for motion tracking. While the HMD might have had a small effect on the acoustics, its impact on the differences in localization performance we investigated was expected to be negligible (Ahrens et al., 2019; Poirier-Quinot & Lawless, 2023). The virtual visual environment rendered by the HMD was implemented in Unity (version 2022.3.0). It showed the inside of a gray sphere representing the world’s boundary, with the participant placed in its center. On that sphere, meridians and horizontal circles were shown for a better orientation along the vertical and horizontal planes, respectively, and the interaural poles were rendered a bit darker to indicate the most lateral directions. The loudspeakers were not rendered. In the display center, a white crosshair was virtually hovering to show the view direction. The frontal anchor position was rendered as a small blue sphere hovering at the frontal direction at the world’s surface. The direction of pointing (controlled by the hand pointer) was rendered as a target circle hovering between the participant and the world’s surface, with the color representing the status of the response: yellow when the stimulus was playing and the response was disabled, green when a response was expected, and red when the response was outside of a specific spatial range. The tracked six degrees of freedom of the hand pointer, combined with the visible rendered pointing direction, enabled the participant to point to elevated directions even with potential biomechanical complications such as limited shoulder mobility. To guide the participant through a trial, additional textual information was displayed at the display’s center whenever required. All components were controlled by a personal computer (64-bit Windows 10, CPU: Intel i7-11700KF 8-core 3.6 GHz, RAM; 16 GB RAM, Graphic card: NVIDIA GeForce RTX 3070 with 8 GB RAM).

The software framework ExpSuite (Mihocic & Majdak, 2020) provided the applications Agra (version 3.3.4) for audiometric measurements, AMT@ARI (version 8.0.5) for HRTF measurements, and LocaDyn (version 0.11.1) for localization experiments.

### 2.4 Procedure

At the beginning of each trial, participants had to fixate the frontal anchor position and confirm via button press. Stimulus playback started after 600 ms. A trial was repeated if the participant’s head orientation deviated more than 5° from the anchor position during stimulus playback. The stimuli were presented from a loudspeaker selected randomly out of a restricted set that was chosen according to previous work (Macpherson & Middlebrooks, 2003): Target positions were mainly placed around the median plane (azimuth range: 0°± 30° and 180°± 30°; elevation range: ± 60°; Figure 2), where spectral cues are most important. Each of those 30 positions was presented twice per block. In addition, there were six positions at the lateral extremes serving as spread controls and motivating the participants to use the entire lateral response range. Those were presented once per block. Other positions were not used during testing but participants were told that sounds may come from all positions, not even restricted to those of the loudspeakers. Participants then indicated the perceived sound direction using the pointer and pressing a button. Then, displayed information guided the participant back to the frontal anchor position, where the next trial was started.

Each trial lasted approximately five seconds, which consisted of the following intervals: pre-stimulus offset of 600 ms, stimulus duration of 250 ms, response time with a median of 1500 ms (IQR from 1034 to 2233 ms), and the period following the response with a median of 2600 ms (IQR from 2179 to 3244 ms). Each block consisted of 66 trials of either the same stimulus (predictable condition) or randomly selected at each trial (unpredictable condition). Participants were not informed whether the stimulus was constant or random; they had to infer this property implicitly by accumulating evidence over successive trials. After each block, a rating of the perceived difficulty was acquired on a five-point Likert scale ranging from ’not at all’ (1) to ’extremely difficult’ (5). Each block lasted approximately six minutes.

Thirteen blocks were tested in total. All participants started and ended with a predictable flat-spectrum block. The remaining eleven blocks (five predictable and six unpredictable) were ordered pseudo-randomly from the residual set, such that no more than two consecutive blocks belonged to the same predictability condition and that the order of predictable and unpredictable conditions was balanced across participants. Breaks between blocks were optional, but mandatory after every third block. Including those breaks, it took participants about 100 minutes to complete the main test.

Before the main test, participants were introduced to the experimental setup and trained until they reached a polar error rate below 5° (qualification criterion). The training procedure was similar to the main test, but used flat-spectrum stimuli presented from all loudspeakers and included a feedback-and-exploration phase: After each response, the actual sound-source location was shown as a red cube and had to be confirmed via a button. This triggered the exploration phase, in which the same stimulus was repetitively played until the participants confirmed the target position again. In this phase, the participant had the chance to explore how the stimulus sounds when moving the head, helping to familiarize themselves with the localization cues. Each training block lasted approximately 20 minutes. One participant required three blocks, another participant two blocks, and all other participants reached the qualification criterion already after a single block.

### 2.5 Data Analysis

Localization performance in the polar angle dimension was evaluated using three error metrics. First, we used the polar error rate metric introduced by Macpherson and Middlebrooks (2003), allowing a direct comparison with their results. The polar error rate quantifies the proportion of responses deviating by more than 45° from a linear regression of flat-spectrum responses (shorter gray lines in Figure 3). The regression lines were fitted to response-target angles separately for targets in front and back per predictability condition and participant. Second, we used the quadrant error rate as another commonly used metric of large-scale localization errors (Middlebrooks, 1999). It is the proportion of errors deviating by at least 90° from the target position (dashed diagonal lines in Figure 3). Compared to the polar error rate, it assesses large errors like front-back confusions, but it does not account for listener-specific systematic response biases observed for flat-spectrum targets. Third, we used the local polar error (Middlebrooks, 1999), defined as the root-mean-square deviation for all responses not flagged as quadrant errors (i.e.,absolute target-response deviations within 90°), and therefore termed “local.” This metric combines accuracy and precision into a single measure of local responses.

**Figure 3.**
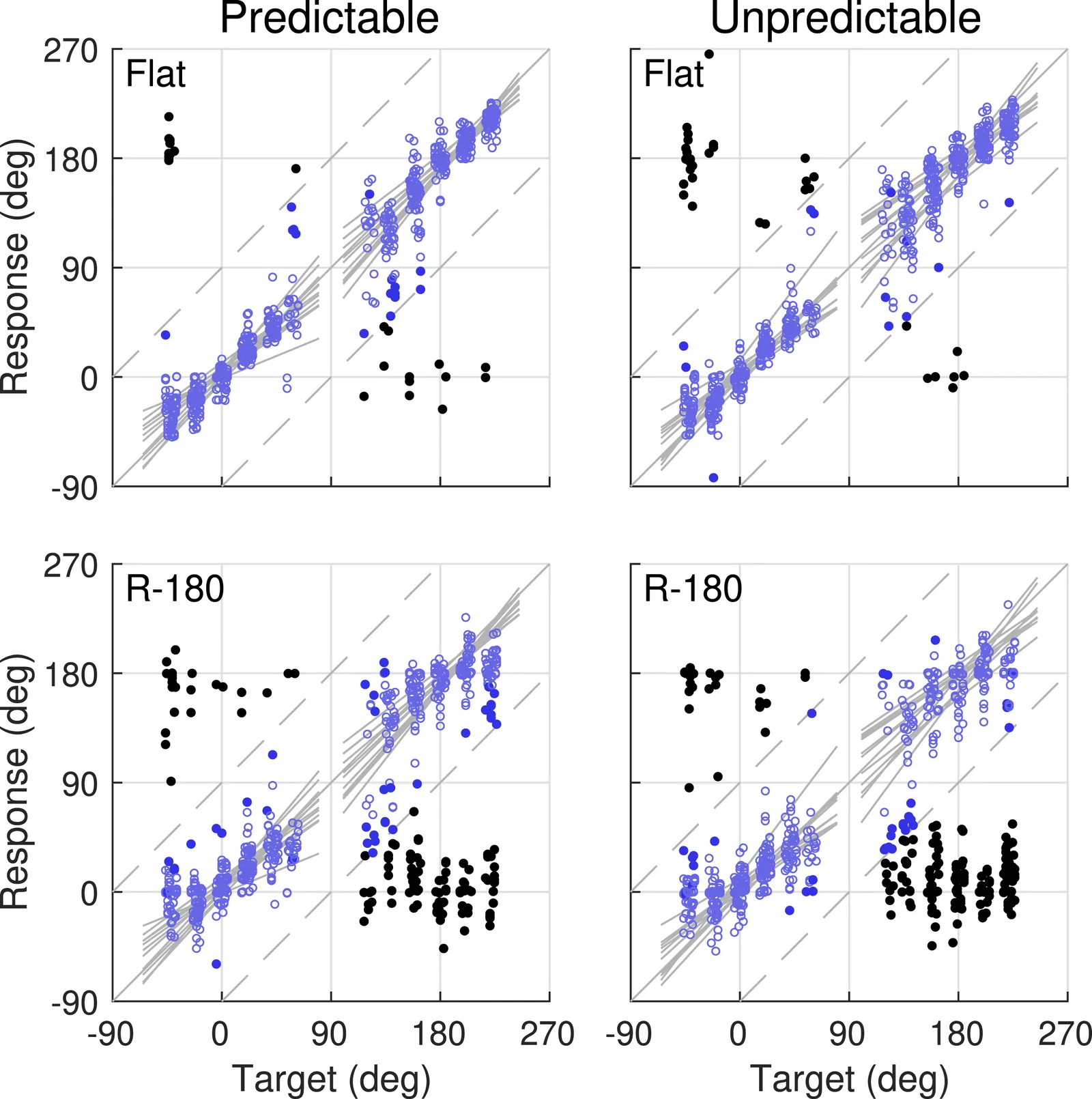
Polar-angle responses for flat-spectrum and R-180 stimuli, shown separately per predictability condition. Responses are classified into three categories per participant: 1) filled black circles: quadrant errors, deviating more than 90° from the main diagonal; 2) blue circles: local responses used to compute the local polar error (RMS deviation across all non-quadrant error responses); 3) filled circles (blue and black): responses deviating more than 45° from the participant’s own regression line, used to determine the polar error rate. Responses from individual participants are horizontally offset for clarity. Short gray lines around main diagonal show baseline regression lines for each participant, determined separately per predictability condition and hemisphere (front and back). Dashed gray lines mark the generic ± 90° quadrant error bounds

Metrics were calculated per condition: stimulus type (flat, R-0, R-45, R-90, R-135, R-180) and predictability (unpredictable, predictable). For the predictable condition, data were obtained from one continuous block. In contrast, for the unpredictable condition, trials from all blocks were sorted by stimulus type. All spread control trials were excluded from further analysis, resulting in 60 data points per participant and condition to compute each metric.

Statistical analyses of the localization metrics were based on a repeated-measures analysis of variance (rmANOVA). The factors included stimulus type and predictability, and analyses were conducted for the polar error rate, quadrant error rate, and local polar error. Residuals were checked for normality, the significance level was set at *α* = 0.05, and the Greenhouse–Geisser correction was applied to all F-tests. Post-hoc comparisons were based on estimated marginal means adjusted using Bonferroni correction. All analyses were performed in RStudio using built-in functions and external toolboxes (Ben-Shachar et al., 2020; Lenth, 2023; Singmann et al., 2023).

To examine potential learning effects, error metrics were also analyzed over the course of blocks. For each participant, responses were grouped across all blocks per predictability condition, yielding six data points per trial index. Metrics were then computed using a sliding window of 20 successive trial indices with a hop size of one trial index. Given an average trial duration of approximately 5 seconds, this window corresponds to about 1.5 minutes of testing time. Finally, a cluster-based permutation test (Gerber, 2014; Maris & Oostenveld, 2007) was applied for each metric to identify time periods within a block showing significant effects of predictability.

## 3 Results

### 3.1 General Localization Performance

Figure 3 shows the polar-angle response patterns pooled across participants for selected conditions. It is evident that large localization errors such as quadrant errors (closed circles) are much less frequent for flat-spectrum sounds (top) as compared to ripple-spectrum sounds (bottom). Such errors also seem to occur slightly less frequently for predictable (left) than unpredictable (right) source stimuli.

Figure 4 shows the performance metrics for each stimulus type grouped by predictability conditions. For the polar error rate, the rmANOVA identified significant main effects for stimulus type, *F*(3.50, 34.95) = 26.71, *p* < .001, *η*^2^ = 0.44, and predictability, *F*(1, 10) = 5.52, *p* = .041, *η*^2^ < 0.01. The interaction between the two factors was not significant, *F*(3.21, 32.10) = 1.42, *p* = .254. A similar pattern emerged for the quadrant error rate: Significant main effects were observed for both predictability, *F*(1, 10) = 19.09, *p* = .001, *η*^2^ = 0.04, and stimulus types, *F*(3.07, 30.74) = 17.06, *p* < .001, *η*^2^ = 0.48, but their interaction was not significant, *F*(2.88, 28.75) = 2.38, *p* = .092. For the local polar error, only stimulus type had a significant effect, *F*(3.19, 31.91) = 46.63, *p* < .001, *η*^2^ = 0.65. Neither predictability, *F*(1, 10) = 0.22, *p* = .646, nor the interaction, *F*(3.16, 31.59) = 0.67, *p* = .583, reached significance.

**Figure 4.**
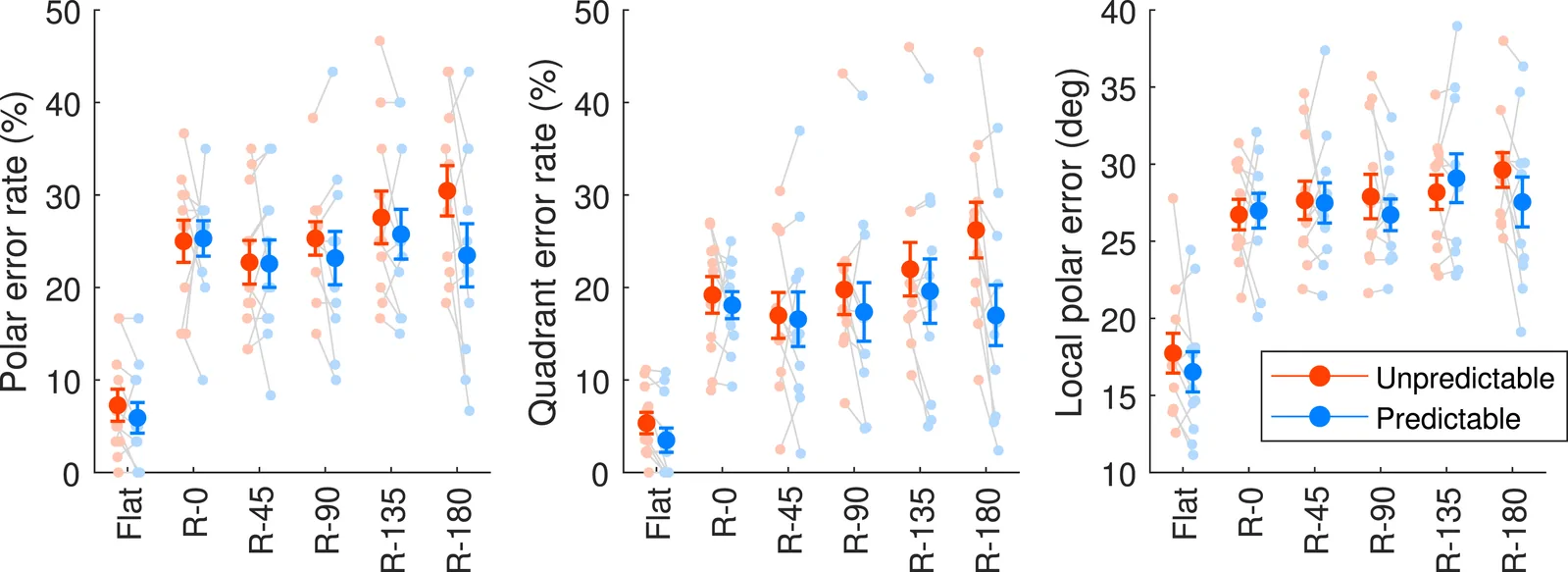
Localization error metrics for each stimulus type grouped by predictability condition. Filled circles and bars show means and standard errors across participants, respectively. Small dots and gray lines show individual participants, with lines connecting unpredictable to predictable conditions for each stimulus type

Post-hoc comparisons confirmed that localization accuracy was consistently higher for *flat-spectrum* stimuli than for *ripple-spectrum* stimuli across all measures. Furthermore, *predictable* conditions yielded fewer localization errors than *unpredictable* ones, particularly in the large-scale error rates. These results suggest that predictability in the source spectrum facilitates some form of adaptation within a block, enabling listeners to resolve spectral ambiguities more effectively.

### 3.2 Temporal Evolution of the Predictability Benefit

To further investigate the time scale of adaptation, we tested whether the predictability benefit evolved over the course of a block, as one would expect in the case of short-term adaptation, or was present to begin with. For this analysis, we collapsed the data across all types of ripple spectra and compared the two predictability conditions at various trial indices within a block. Figure 5 shows the temporal evolution for the three localization error metrics. As expected, predictability had no effect on localization performance within the first trails of a block. After almost 40 trials, that is, about 3 to 4 minutes into the block, predictability led to a consistent improvement in the large-scale error rates, as confirmed by cluster-based permutation testing (polar error rate: *t*_*cum*_ = 81.95, *p* = .005; quadrant error rate: *t*_*cum*_ = 58.35, *p* = .011). No significant differences were found for the local polar error rate (*t*_*cum*_ = 11.59, *p* = .226), consistent with the temporally unresolved analyses reported above. However, the time courses suggest a tendency toward improvement specific to the predictable condition, which may not have reached statistical significance in the permutation test because the local polar error metric was not equivalent at block onset.

**Figure 5.**
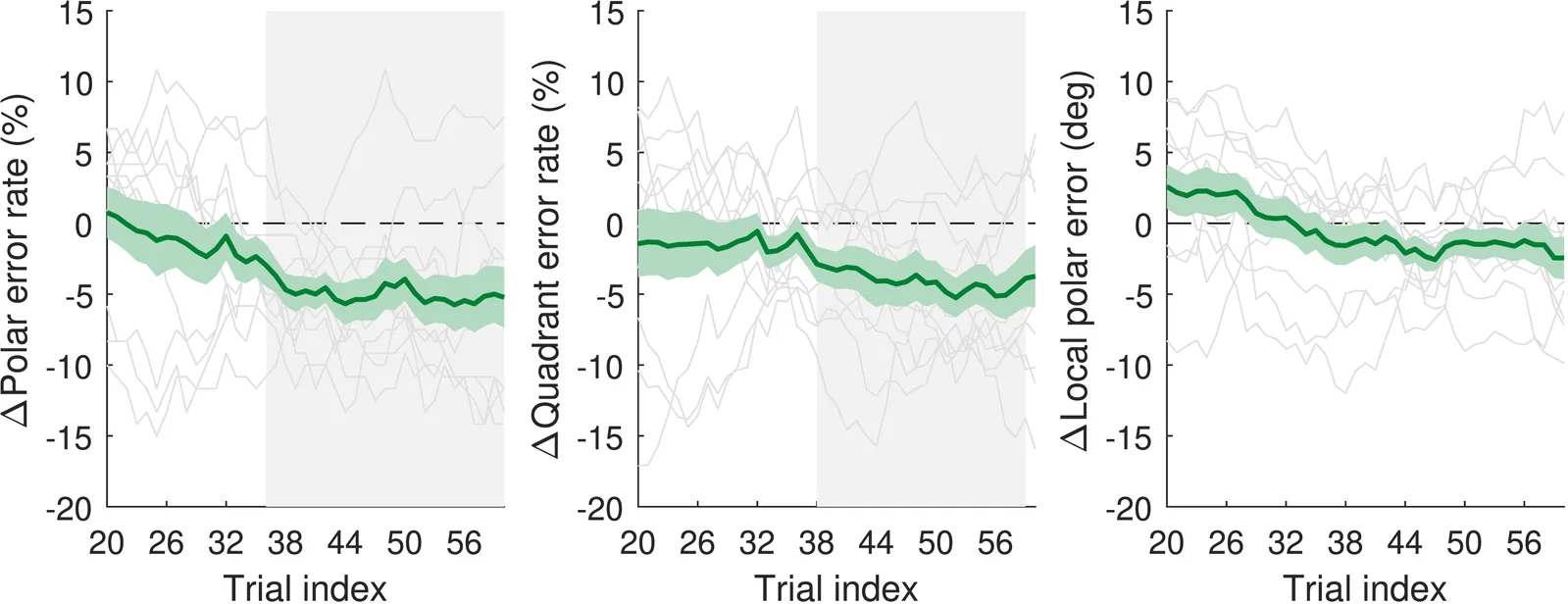
Temporal evolution of the predictability effect (predictable minus unpredictable) for each localization error metric. Thin gray lines show individual participants. Dark green line and shaded area show the mean and standard error across participants, respectively, calculated over a sliding window of 20 trials. Dashed line marks zero (no effect). Gray-shaded areas indicate trial indices where the difference between conditions was statistically significant (cluster-based permutation test, *p* < 0.05)

The curves shown in Figure 5 indicate that the predictability benefit for ripple-spectrum sounds seems to plateau around 5% for both large-scale error rates. In comparison, the difference between flat- and ripple-spectrum sounds was more in the order of 15%. Hence, the short-term adaptation was not able to completely overcome the interference caused by the spectral ripples.

### 3.3 Difficulty Ratings

Participants rated both the predictable and unpredictable conditions as moderately difficult (“somewhat difficult”; Figure 6). No significant difference emerged between conditions (Wilcoxon paired rank-sum test, *p* > .05), although ratings tended to be lower for the predictable condition, consistent with the performance benefit observed at the end of predictable blocks.

**Figure 6.**
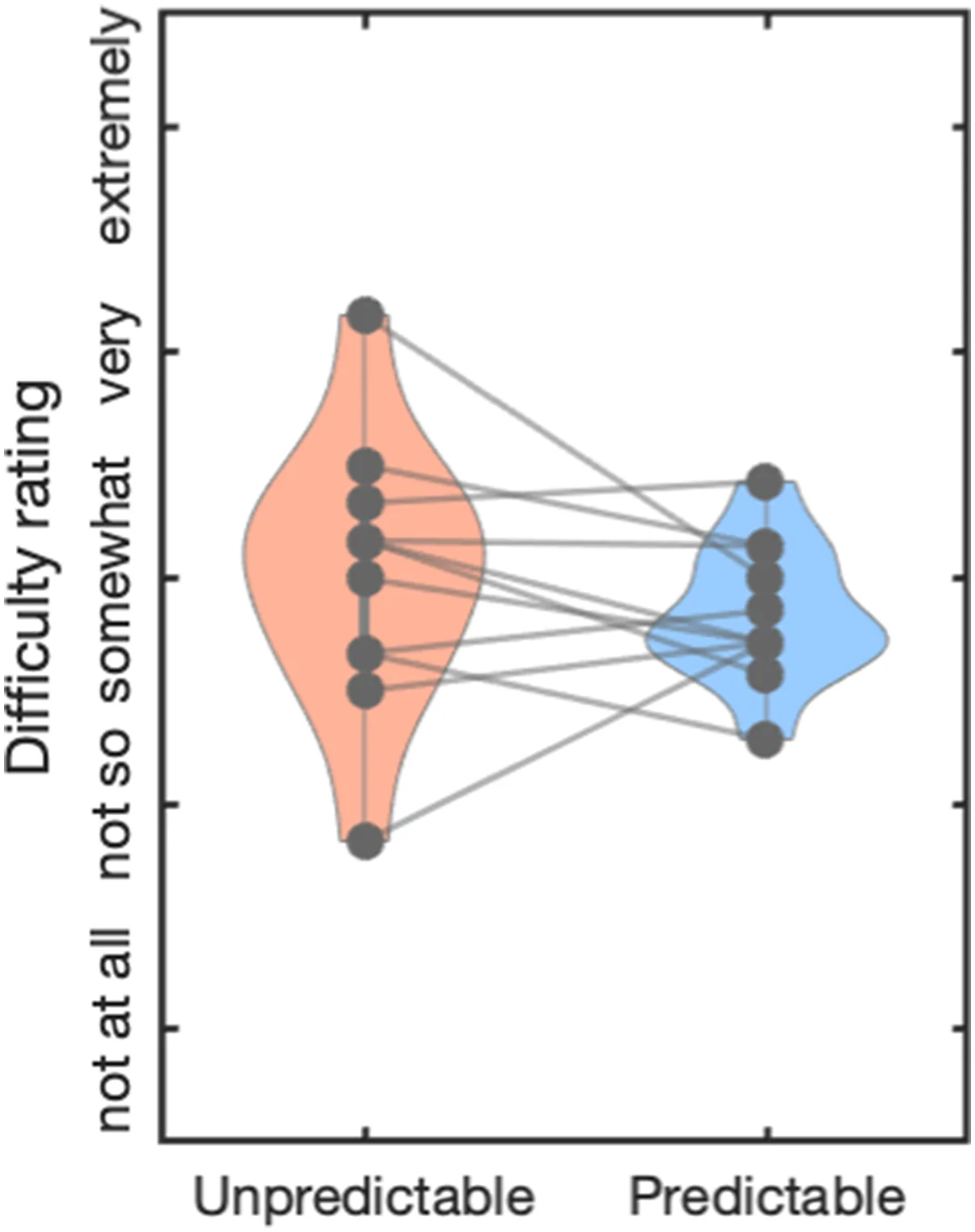
Difficulty ratings averaged across stimulus types. Violin plots show estimated distributions of ratings across participants. Individual participant’s averages are connected by horizontal lines

## 4 Discussion

Participants adapted to the spectral characteristics of sound sources, using this information to improve localization performance. Ripple-spectrum stimuli introduced strong spectral interference and degraded performance compared to flat-spectrum stimuli. However, when the source spectrum remained fixed across trials – unknown a priori – listeners reduced large-scale localization errors over tens of trials. Improvements in the local error metric and perceived task difficulty were not significant, though trends aligned with the effects found for the global error metrics. Together, these results support the hypothesis that the auditory system implicitly exploits short-term spectral consistency to constrain spatial inference and mitigate the ill-posed nature of localization from spectral cues – even without explicit feedback.

The unpredictable condition was intended to replicate findings from Macpherson and Middlebrooks (2003), where polar error rates increased from about 5% for flat-spectrum stimuli to 34% for ripple-spectrum sounds. In that study, the ripples were always fixed within each block, but interleaved with flat-spectrum reference targets, so the unpredictable condition here is more unpredictable than that. Nevertheless, our results show a similar trend, with errors rising from 7% to 26%. Like Macpherson and Middlebrooks (2003), we observed substantial variability across listeners, particularly across ripple phases. These interindividual differences likely reflect idiosyncratic HRTFs (Majdak et al., 2014; Middlebrooks, 1999). Overall, these findings confirm that ripple-spectrum noise reliably degrades localization performance.

Those ripple-spectrum sounds were chosen to maximally interfere with localization cues. Natural sounds like speech are known to cause less interference (Best et al., 2005). In part, this is due to less pronounced spectral modulations at high frequencies in combination with spectral gradient extraction mechanisms that increase robustness to low spectral modulation rates (Barumerli et al., 2023; Baumgartner et al., 2014; Zakarauskas & Cynader, 1993). In addition, long-term ecological priors may account for improved sound localization of such natural sources (Reijniers et al., 2025).

Although our auditory system may utilize long-term priors for natural sounds, our study shows that listeners are able to adapt even to the highly unnatural ripple-spectrum sounds – if presented consistently within a block. Our listeners improved their localization performance within approximately 40 trials, that is 3 minutes from the beginning of a block. Similar adaptation rates were previously observed in auditory brightness perception (Siedenburg et al., 2021) and such a similarity between timbre and space is particularly interesting in regards of potential interactions between cues encoding source identity and position.

Evidence for interactions in processing space and identity information is mixed. On one hand, cortical processing is widely understood to be largely segregated into the ventral “what” and dorsal “where” auditory pathways (Ahveninen et al., 2006; Bizley & Cohen, 2013; Majdak et al., 2020). Additionally, Rakerd et al. (1999) provided specific counter-evidence for space-identity interactions. In their study, they conducted parallel experiments on the identification and localization of sounds in the median plane, demonstrating that listeners can localize noises they cannot identify and vice versa.

On the other hand, perceptual inference likely involves interactions between the two pathways. Attention and working memory may dynamically bias the weighting of identity versus spatial cues, depending on task relevance and the perceived stability of the acoustic environment (Gazzaley & Nobre, 2012). These biases can also be shaped by ecological significance and threat-related processing. For example, a recent study showed that looming sounds – associated with approaching objects – preferentially engage frontal regions linked to threat detection and bias processing within the ventral “what” stream, even when the task focuses solely on movement-direction discrimination (Ignatiadis et al., 2025). Such findings indicate that perceptual weighting is not only task-dependent but also influenced by learned or innate priors about environmental relevance.

These dynamic weighting processes align with the Bayesian perspective on auditory inference, which formalizes cue integration and prior updating. Bayesian frameworks for sound localization rely on probabilistic integration of sensory cues and prior expectations (Barumerli et al., 2023; Ege et al., 2019). However, most models assume a broadband flat spectrum and treat spectral information primarily as spatial information. Recently, an ideal-observer model was proposed for localizing sound sources with unknown spectra by deriving ecologically valid priors from environmental sounds and speech (Reijniers et al., 2025). This model suggests that the auditory system applies generic spectral priors to resolve ambiguities. Our findings extend this perspective by showing that listeners can also update these priors on short timescales, indicating coexistence of long-term priors and short-term updates. Incorporation of such dynamics in Bayesian inference frameworks (e.g., Barumerli & Majdak, 2025) may open new avenues for computational modeling of auditory perception.

However, our findings also raise questions about the limits of short-term adaptability, which future studies should address. In our study, the benefit of predictability was considerably smaller than the distortion introduced by stimulus type (flat vs. rippled spectra). Similarly, modest benefits of predictability were also found previously (chapter 3,Macpherson, 1998). One possible explanation is that the artificial nature of ripple spectra introduces particularly high uncertainty about the source identity. This could be tested by contrasting adaptability between natural and artificial sources or by reducing identity uncertainty – for example, by presenting the target’s spectrum at the start of each block. Another factor may be inter-stimulus intervals, which in our study were on the order of seconds. Shorter intervals strengthen stimulus-specific adaptation (Briley & Krumbholz, 2013) and may therefore enhance statistical learning, helping to mitigate the ill-posed problem of sound localization.

## 5 Conclusions

Our findings demonstrate that the auditory system can rapidly exploit short-term spectral consistency to improve localization, even under highly artificial conditions. This supports the view that spatial inference is not solely governed by long-term priors but dynamically integrates recent sensory history to reduce ambiguity. By revealing rapid adaptation within minutes, our study bridges ecological models of auditory perception with experimental evidence and underscores the flexible interplay between identity and spatial cues. These insights advance theoretical frameworks of auditory inference and inform practical applications – from hearing-aid algorithms to immersive virtual environments – where accurate spatial audio rendering is essential.

## Data Availability

Localization responses and listener-specific HRTFs (recorded but not used for present study as sounds were presented via loudspeakers) can be downloaded (Baumgartner et al., 2026) and will be available for computational modeling as data_baumgartner2026a together with the stimulus generation code sig_macpherson2003 within the AMT (Majdak et al., 2022) version 1.7.0 (Majdak et al., 2026).
